# Supplementary material for: The impact of pathological high-frequency oscillations on hippocampal network activity in rats with chronic epilepsy
Source: eLife. 2019 Feb 22;8:e42148. doi: 10.7554/eLife.42148 (PMC6386518; doi:10.7554/eLife.42148)
Supplement: Figure 6—source data 1. — Neurons are grouped according to whether they are modulated by ripple, ripple-like, or pHFO events. These measures are calculated per foraging periods, such that N reflects the number of neurons multiplied by the number of foraging periods. Values are reported as median; inter-quartile range. [file elife-42148-fig6-data1.docx]

**Table 1. Spatial coding parameters of place cells in control and epileptic animals.** Neurons are grouped according to whether they are modulated by ripple, ripple-like, or pHFO events. These measures are calculated per foraging period, so the N reflects the number of neurons multiplied by the number of foraging periods. Values are reported as median; inter-quartile range.

|  | Control | Epilepsy |  |
| --- | --- | --- | --- |
|  | Ripple Modulated n = 87 | Ripple-Like Modulated n= 36 | pHFO Modulated n= 27 |
| Spatial Information (bits) | 1.6; 1.1 – 2.2 | 0.5; 0.3 – 1.1 | 0.5; 0.2 – 0.7 |
| Within Session Correlation | 0.9; 0.7 – 0.9 | 0.3; 0.1 – 0.7 | 0.3; 0.1 – 0.6 |
| Spatial Sparsity | 0.2; 0.2 – 0.4 | 0.6; 0.3 – 0.7 | 0.5; 0.5 – 0.8 |
